# P-1120. Antibiotics use among premature infants in three neonatal care Units in Brazzaville, Republic of Congo

**DOI:** 10.1093/ofid/ofae631.1307

**Published:** 2025-01-29

**Authors:** Martin Herbas Ekat, Rolland Bienvenu Ossibi Ibara

**Affiliations:** Brazzaville University Hospital, Brazzaville, Brazzaville, Congo, (Congo Â– Brazzaville); Brazzaville University Hospital, Brazzaville, Brazzaville, Congo, (Congo Â– Brazzaville)

## Abstract

**Background:**

To evaluate the use of antibiotics in Congolese premature infants based on WHO recommendations in three neonatal care units in Brazzaville.

**Methods:**

This was a cross-sectional study, premature infants admitted 72 hours after birth in three neonatals care units in Brazzaville, between January 2019 and November 2020, were included. Taking into account WHO recommendations, we grouped premature infants according to the presence or absence of maternal risk factors for neonatal infection (3 items), and each factor was rated 1. Risk factors for neonatal infection, such as early onset sepsis (EOS) specific to the newborn (7 items), were also used and rated 1 or 0 depending on whether it was present or not. Maternal risk had 4 categories (0, 1, 2 and 3) and EOS risk had 8 categories (0, 1, 2, 3, 4, 5, 6 and 7). Premature newborns were first classified according to maternal risk followed by EOS risk for each maternal risk category, then the proportion of premature infants placed on antibiotics was determined.

**Results:**

Aa total of 448 premature newborns were included. The proportion of antibiotic therapy depending on the categories was as follows: maternal risk 3 (EOS risk 2, 3 and 5 were 100% and INBP risk 4 67%), maternal risk 2 (INBP risk 1, 2, 3, 4 and 5: 0%, 64%, 92%, 91% and 100% respectively), maternal risk 1 (EOS risk 1, 2, 3, 4, 5 and 6: 88%, 66%, 88%, 93%, 100% and 100% respectively), maternal risk 0 (INBP risk 1, 2, 3, 4, 5 and 6: 29%, 57%, 76%, 82%, 96% and 100% respectively). In multivariate analysis, the factors associated with the use of antibiotic therapy outside the WHO recommendations were: being born in a health structure different from that of hospitalization (OR: 0.16; 95%CI: 0.03 – 0.94 ), be born in an integrated health center (OR: 23; 95%CI: 2.9 – 190).

**Conclusion:**

The Use of antibiotic therapy is only effective when all the maternal or premature newborn risk factors are present, contrasting with WHO recommendations. Other factors are involved in the use of antibiotics, such as being born in primary healthcare facility. The implementation of an antibiotic management program is a necessity.

**Disclosures:**

**All Authors**: No reported disclosures

